# Will ocean acidification affect the early ontogeny of a tropical oviparous elasmobranch (*Hemiscyllium ocellatum*)?

**DOI:** 10.1093/conphys/cow003

**Published:** 2016-03-04

**Authors:** Martijn S Johnson, Daniel W Kraver, Gillian M C Renshaw, Jodie L Rummer

**Affiliations:** af1 ARC Centre of Excellence for Coral Reef Studies, James Cook University, Townsville, Queensland 4811, Australia; af2 College of Marine and Environmental Science, James Cook University, Townsville, Queensland 4811, Australia; af3 Hypoxia and Ischemia Research Unit, School of Allied Health Science, Griffith University, Gold Coast, Queensland 4222, Australia

**Keywords:** Elasmobranch, embryonic development, mesopredator, ocean acidification, oviparous

## Abstract

Atmospheric CO_2_ is increasing due to anthropogenic causes. Approximately 30% of this CO_2_ is being absorbed by the oceans and is causing ocean acidification (OA). The effects of OA on calcifying organisms are starting to be understood, but less is known about the effects on non-calcifying organisms, notably elasmobranchs. One of the few elasmobranch species that has been studied with respect to OA is the epaulette shark, *Hemiscyllium ocellatum*. Mature epaulette sharks can physiologically and behaviourally tolerate prolonged exposure to elevated CO_2_, and this is thought to be because they are routinely exposed to diurnal decreases in O_2_ and probably concomitant increases in CO_2_ in their coral reef habitats. It follows that *H. ocellatum* embryos, while developing *in ovo* on the reefs, would have to be equally if not more tolerant than adults because they would not be able to escape such conditions. Epaulette shark eggs were exposed to either present-day control conditions (420 µatm) or elevated CO_2_ (945 µatm) and observed every 3 days from 10 days post-fertilization until 30 days post-hatching. Growth (in square centimetres per day), yolk usage (as a percentage), tail oscillations (per minute), gill movements (per minute) and survival were not significantly different in embryos reared in control conditions when compared with those reared in elevated CO_2_ conditions. Overall, these findings emphasize the importance of investigating early life-history stages, as the consequences are expected to transfer not only to the success of an individual but also to populations and their distribution patterns.

## Teaser

Ocean acidification is predicted to negatively affect marine organisms, but little is known regarding effects on elasmobranchs, especially early in development. Neither growth, yolk consumption, ventilation, nor movement were affected in epaulette shark embryos reared under end of-century CO2 conditions, suggesting that tolerance may start very early in life, just post-fertilization.

## Introduction

Global industrialization, rapid deforestation and the burning of fossil fuels as a primary energy source have caused an increase in the amount of anthropogenic CO_2_ released into the environment. Much of the excess CO_2_ is absorbed into the oceans, causing significant changes in the oceans' natural carbonate systems and, most notably, ocean acidification (OA; Millero, 2007; Doney *et al*., 2009b). The ongoing process of OA poses a serious threat to many marine species and ecosystems and has therefore become a paramount focus of study (Hendriks *et al*., 2010; Kaniewska *et al*., 2012). The ocean has absorbed ∼30% of excess atmospheric CO_2_ since the industrial revolution, which has resulted in a decrease in pH of ∼0.1 units. This is an increase in partial pressure of CO_2_ (pCO_2_) from pre-industrial levels of 200–280 µatm (Feely *et al*., 2004; Solomon *et al*., 2009) to present-day levels, which are already exceeding 400 µatm (Trans and Keeling, 2015). If this trend continues, it is predicted that pCO_2_ will increase to ∼1000 µatm, and pH will decrease by a further ∼0.3–0.4 units by the end of century, thus increasing ocean acidity by ∼150% from the beginning of the industrial revolution (Orr *et al*., 2005; Doney *et al*., 2009b; Feely *et al*., 2009; Kelly *et al*., 2012). It is predicted that many marine species will be negatively affected by OA, and this has been especially evident in calcifying organisms (Kleypas *et al*., 1999; Riebesell *et al*., 2000; Feely *et al*., 2004; Orr *et al*., 2005; Kurihara, 2008; Gooding *et al*., 2009; Barton *et al*., 2012). However, there is a paucity of information regarding non-calcifying organisms, especially elasmobranchs, despite evidence that some elasmobranch families may be more sensitive to climate change than others (Chin *et al*., 2010; Dulvy *et al*., 2014).

Sharks, rays and skates (subclass elasmobranchii) are considered some of the most vulnerable of all marine vertebrates. Indeed, nearly a quarter of all elasmobranch species are threatened by extinction (Lisney *et al*., 2012; Ward-Paige *et al*., 2012; Dulvy *et al*., 2014). Their life-history characteristics contribute to their sensitivity, as adults mature slowly and exhibit low fecundity that results in very few offspring that undergo a long gestation time, all of which increases population-level sensitivity. Anthropogenic stressors, such as habitat degradation, overfishing, bycatch, pollution and climate change, add to their vulnerability (Ellis *et al*., 2004; Simpfendorfer and Heupel, 2004; Ebert *et al*., 2008; Flewelling *et al*., 2010; Renshaw *et al*., 2012; Ward-Paige *et al*., 2012; Rosa *et al*., 2014).

There are knowledge gaps and relatively few studies addressing the direct effects of elevated CO_2_ on elasmobranchs. A reason for the paucity may be because elasmobranchs are not expected to be physiologically vulnerable to elevated CO_2_. The animals that would eventually become modern elasmobranchs evolved ∼420 million years ago, when atmospheric CO_2_ was eight to 10 times greater than it is today (Clack, 2007). Therefore, it is thought that elasmobranchs (like the bony fishes) already possess the HCO_3_^−^ exchange mechanisms necessary to regulate acid–base/ions efficiently in the presence of CO_2_ levels similar to those predicted for the end of the century (Claiborne and Evans, 1992; Brauner and Baker, 2009; Tresguerres *et al*., 2010). Recent studies, however, have demonstrated numerous negative effects of elevated CO_2_ on elasmobranchs. Dixson *et al*. (2015) found that in conditions of elevated CO_2_, smooth dogfish (*Mustelus canis*) did not respond to odour cues indicative of food, suggesting that food foraging may be affected in some elasmobranchs. This finding is also supported in the mesopredator Port Jackson shark (*Heterodontus portusjacksoni*), in which elevated CO_2_ negatively affected hunting behaviour (via olfaction) as well as growth and metabolic efficiency (Pistevos *et al*., 2015). Negative effects of elevated CO_2_ (pH 7.5) and warmer temperatures (+3°C above average) have been documented in pre- and post-hatching survival of brown-banded bamboo shark (*Chiloscyllium punctatum*) embryos as well (Rosa *et al*., 2014). Temperate water little skate (*Leucoraja erinacea*) embryos also increase their cost of activity and development time and reduce body condition when reared in elevated CO_2_ (pH 7.7) and at temperatures that are 3–5°C above average (Di Santo, 2015). The effects of climate change-relevant levels of elevated CO_2_ and ocean acidification on elasmobranchs are still underexplored, but the few studies that have been conducted have definitely highlighted the need for more research.

Many elasmobranchs have the ability to move to a more suitable habitat when their environment becomes unfavourable (Speed *et al*., 2010; Simpfendorfer *et al*., 2011; Papastamatiou and Lowe, 2012). However, this may not be the case during certain developmental stages. Oviparous elasmobranchs (those that develop in eggs outside of the mother's body) are restricted to wherever the eggs are deposited on the benthos for the duration of embryonic development (Amsler *et al*., 2015). This means that, during embryonic development, the animal cannot move if environmental conditions become adverse (Rodda and Seymour, 2008). Furthermore, no maternal care is given to elasmobranch eggs; once deposited, embryos remain in position until hatching and are thus in constant contact with the local environment (Hamlett, 2005; Musick and Heithaus, 2012). Egg cases are made of fibrous layers and have a leathery texture, ensuring that the delicate embryos inside have some protection against the elements, physical damage and pathogens (Lucifora and Garcia, 2004; Rodda and Seymour, 2008). However, there is active water exchange between the external environment and the internal environment of the egg, and therefore, the egg case does not shelter the embryo from changes in water chemistry (Rodda and Seymour, 2008). Thus, small, bottom-dwelling elasmobranchs that use this type of reproductive/development mode may be at risk from changes in water quality associated with climate change during embryonic development.

Epaulette sharks (*Hemiscyllium ocellatum*) live on shallow coral reef platforms on the Great Barrier Reef, where they routinely experience low O_2_ levels during nocturnal low tides (Wise *et al*., 1998; Nilsson and Renshaw, 2004) and have been demonstrated to be hypoxia (Routley *et al*., 2002) and anoxia (Renshaw *et al*., 2002) tolerant. In fact, *H. ocellatum* is one of the few anoxia-tolerant elasmobranchs described to date. These reef flats also experience diurnal increases in pCO_2_ nearing 1000 µatm, and levels in caves, crevices and tide pools, frequently inhabited by epaulette sharks could exhibit even higher CO_2_ levels (Last and Stevens, 2009; Shaw *et al*., 2013). Two recent studies have documented that *H. ocellatum* are able to maintain physiological performance and normal feeding and sheltering behaviours in the presence of elevated CO_2_ (Heinrich *et al*., 2014, 2015). It follows that, if adults are tolerant to low O_2_ and elevated CO_2_, then embryos developing *in ovo* and newly hatched neonates may also be as tolerant, if not more tolerant than adults, because embryos are confined to their egg cases during early development. We hypothesized that the early ontogeny of *H. ocellatum* will not be affected by prolonged exposure to elevated CO_2_ because they possess this capacity for maintaining physiological performance and behaviour as seen in adults exposed to elevated CO_2_ or low O_2_ (Wise *et al*., 1998; Nilsson and Renshaw, 2004; Heinrich *et al*., 2014, 2015). This research aims to provide information on the early ontogeny of epaulette sharks and their developmental tolerance or intolerance to elevated pCO_2_, by comparing embryos reared in present-day (control) conditions with those reared in simulated end-of-century ocean acidification (elevated CO_2_) conditions. Our findings may unmask the effect of an anthropomorphic selection pressure that is important for assessing their adaptive capacity under climate change.

## Materials and methods

### Care of animals

*Hemiscyllium ocellatum* embryos were sourced from two different locations, Sea World Gold Coast in Southport (*n* = 17) and Cairns Marine (*n* = 29), both in Queensland, Australia. Fertile eggs were supplied within 10 ± 2 days of being deposited (days post-fertilization; dpf). Once collected, eggs were transported inside insulated boxes (in oxygen-filled plastic bags containing seawater) via air to Townsville, Queensland, Australia. Upon transfer to the Marine Aquaculture Research Facilities Unit at James Cook University, the egg cases were submerged in dechlorinated freshwater for 2 min as a prophylactic and then placed in a recirculating filtered and ultraviolet-sterilized seawater system (45 l aquaria) in fixed conditions (28.8°C, 8.14 pH) that match present-day conditions for this part of the Great Barrier Reef. Egg cases were suspended vertically, ∼5 cm below the water surface, using plastic pegs and then left undisturbed as a quarantine procedure (7 days). Following this period, individual egg cases were randomly assigned to control tanks (*n* = 16, divided between three aquaria) or elevated CO_2_ tanks (*n* = 20, divided between three aquaria) with no more than five eggs per tank at any time (Table 1).

**Table 1:** Means ± SD for partial pressure of CO_2_, pH (National Bureau of Standards Scale; pH_NBS_), total alkalinity, salinity and temperature for control and elevated-CO_2_ aquaria

Treatment	Partial pressure of CO_2_ (μatm)	pH_NBS_	Total alkalinity (μmol kg^−1^)	Salinity (ppt)	Temperature (°C)
Control, *n* = 16	422.61 ± 45.74	8.14 ± 0.03	2151.53 ± 53.31	35.09 ± 1.12	28.49 ± 0.35
Elevated CO_2_, *n *= 20	945.40 ± 131.09	7.88 ± 0.05	2323.20 ± 82.63	33.58 ± 1.37	28.29 ± 0.18

### Carbon dioxide manipulation and experimental conditions

Two 8000 l recirculating seawater systems were used to achieve desired water quality parameters in all aquaria, each simulating either present-day (control, ∼400 μatm) or predicted end-of-century (elevated CO_2_, ∼945 μatm) conditions (Table 1). Carbon dioxide levels were achieved and maintained by CO_2_ infusion of seawater in 3000 l sumps that were attached to each system (as per Heinrich *et al*., 2014, 2015). The pH_NBS_ (National Bureau of Standards Scale) was set to match present or future CO_2_ concentrations and maintained by an Aqua Medic AT Control System (Aqua Medic, Germany). If pH rose above the allocated set point, a solenoid initiated the system to deliver a steady stream of CO_2_ into the sump. The central approach to the pH manipulation allowed for stability in seawater pH and pCO_2_. The pH_NBS_ was taken daily using a pH electrode (SevenGo Pro; Mettler Toledo, Switzerland). Temperatures of each treatment were maintained using automated chillers and heaters attached to each system and monitored with a standard temperature probe (Comark C26, Norwich, UK). Salinity was measured weekly. Dissolved oxygen was continuously monitored, and throughout the duration of the study neither system fell below 90% air saturation. Nitrogenous waste removal was achieved via activated carbon and biological filtration, and ammonia levels never exceeded 1 ppm. Total alkalinity (TA) was estimated weekly using gran titrations and certified reference materials (Dr A. G. Dickson, Scripps Institution of Oceanography). Average seawater pCO_2_ was calculated using these parameters in CO2SYS (Pierrot *et al*., 2006) using constants from Dickson and Millero (1987).

### Measurements and observations

**Figure 1:**
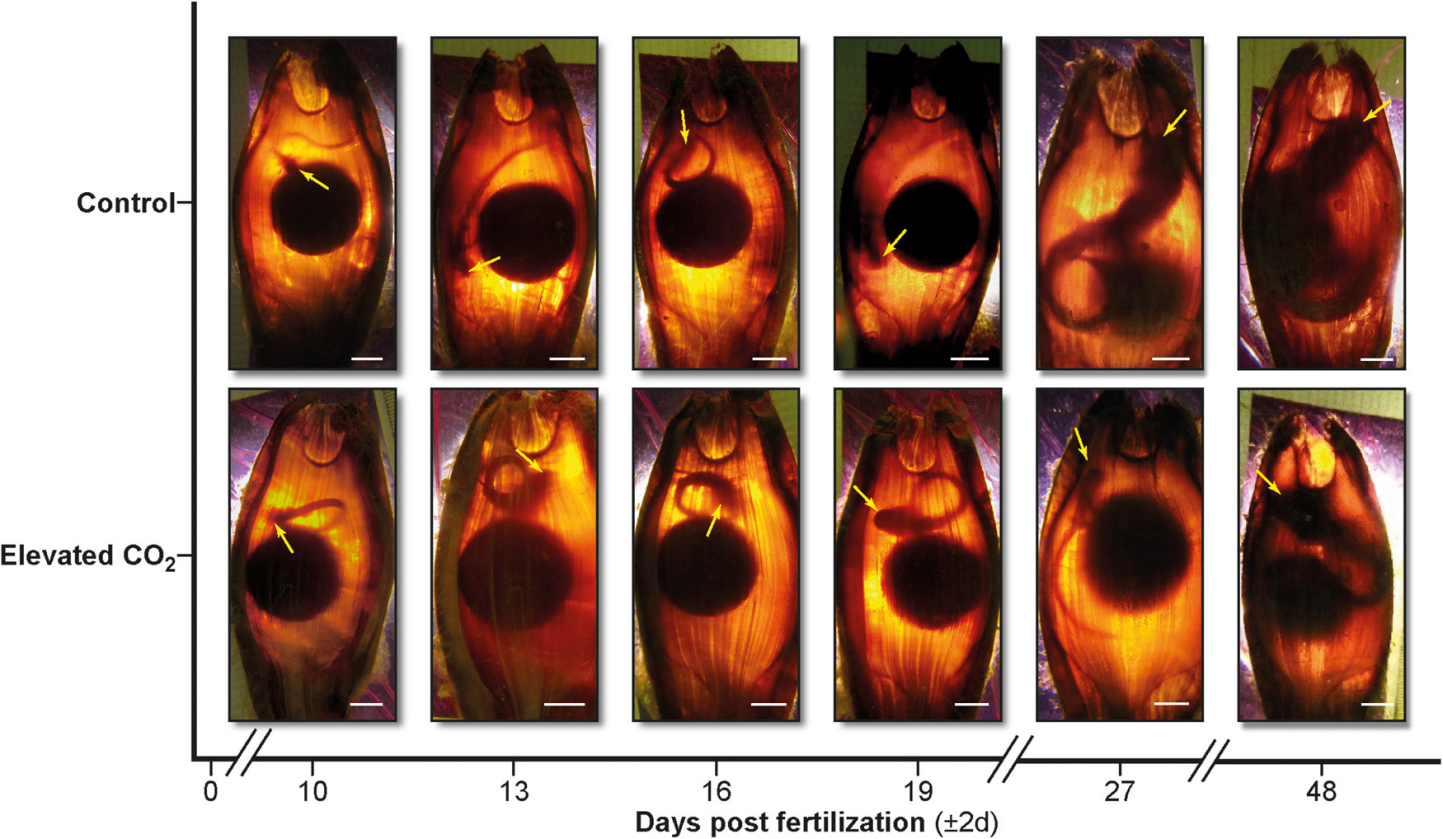
Progression of growth using candling techniques in embryos reared in control (420 μatm) and elevated CO_2_ (940 μatm) conditions from 10 to 48 days post-fertilization (dpf). Yellow arrows point to the embryo's head, and the scale bar in each image is 1 cm.

After the quarantine period, the opaque fibrous layer of each egg was removed using a sterile surgical scalpel, which ensured better resolution for photographs and video. This has been previously shown not to interfere with normal developmental processes (Harahush *et al*., 2007). Thereafter, embryos and yolk were easily seen when placed against a light source, a technique known as ‘candling’. Candling was conducted every 3 days for each egg. To do this, individual eggs were removed from their holding aquaria and placed in a 6 l aquarium containing their respective water treatment and positioned next to a ruler (Fig. 1). A ∼400 lumen torch was placed below the tank to illuminate the embryo through the egg case.

Key developmental markers were monitored using photographs and videos every 3 days. Photographs were taken and analysed (FIJI; Schindelin *et al*., 2012) to determine the average surface area of the embryo and the yolk. Videos were recorded to calculate ventilation rates (per minute) and tail oscillation rates (per minute) for each animal and examined in QuickTime media player (Apple Inc., Cupertino, CA, USA). Data were also collected upon hatching to document the time to hatching and post-hatching survival.

### Calculations and statistical analyses

The surface area of both the embryo and the yolk were calculated every 3 days from three separate photographs of the same individual and reported as the average of the three measurements. This technique was used to determine daily growth rate (in square centimetres per day) and daily yolk consumption, reported as a decrease from 100% over the development period. Videos allowed for the calculation of ventilation rates and tail oscillation rates (per minute). Mixed linear models were used to determine whether there were any significant differences between animals reared control and elevated CO_2_ conditions. Data were presented as means with 95% confidence intervals, and significant differences between control and elevated CO_2_ embryos were reported for each of the following measurements: (i) proportional growth over time; (ii) percentage of yolk consumed over time; (iii) ventilation rates; and (iv) tail oscillation rates.

Numerous mixed linear models were run, and a model was chosen when results showed heterogeneous variances. Tank number and egg number were used as group variables and random effects, whereas treatment (control and elevated CO_2_), origin (Sea World and Cairns Marine) and dpf were treated as independent variables and fixed effects, which accounted for any tank, treatment, origin and time differences, as well as any interactions. A χ^2^ contingency test and Student's unpaired *t*-test were used to examine whether there was a significant difference in the hatching success of embryos in each treatment (control vs. elevated CO_2_) grouped by individual tanks. Significance was measured to an α level of 0.05, and all statistical tests were executed using SPLUS (Insightful, Seattle, WA, USA).

## Results

### Embryo growth and development

**Figure 2:**
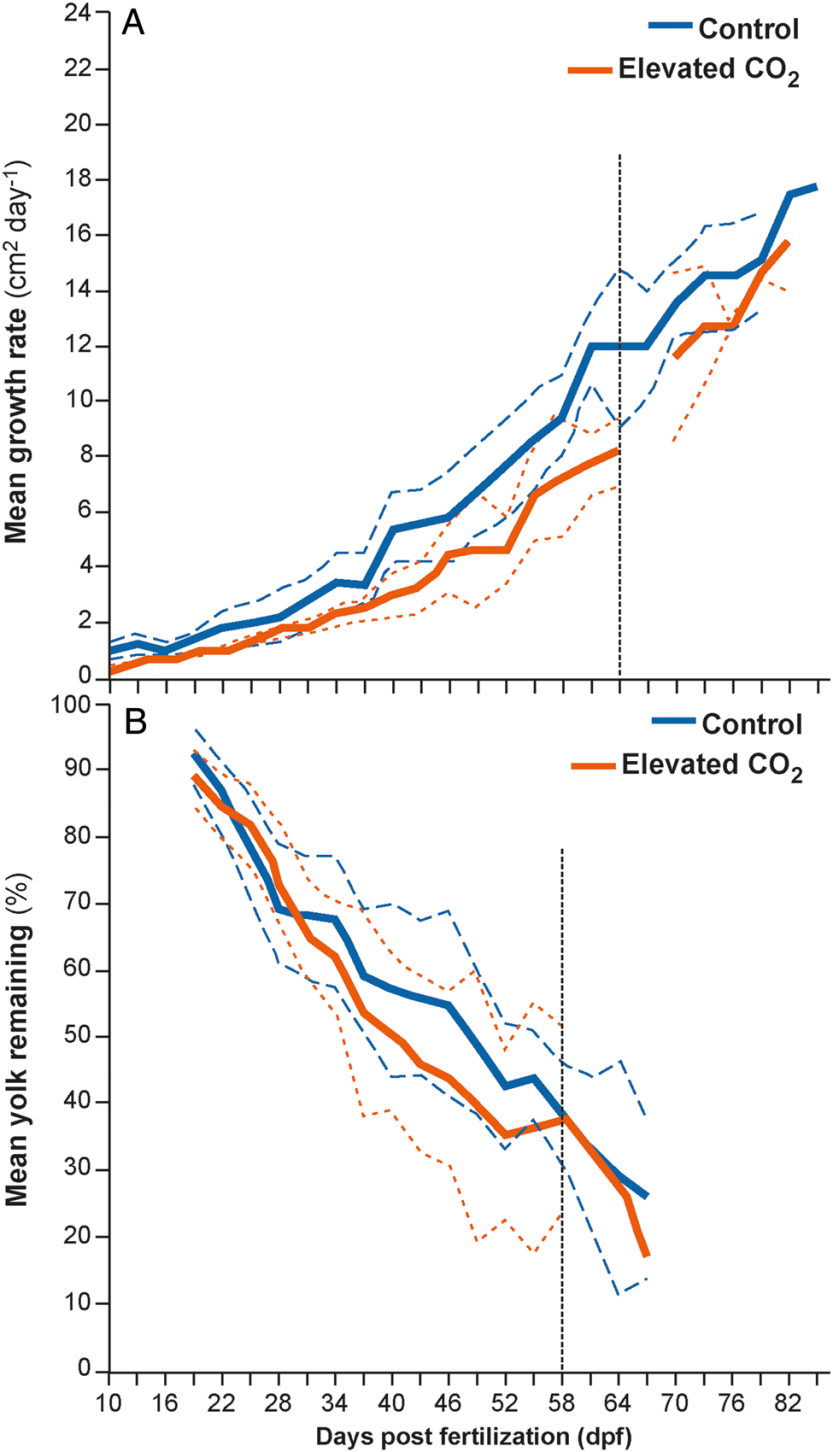
Mean (95% confidence interval) daily growth rate of embryos as expressed as surface area (in square centimetres per day; **A**) and mean (95% confidence interval) yolk remaining in embryos (from 100%; **B**) for control (420 μatm, blue; *n* = 16) and elevated CO_2_ treatment (940 μatm, orange; *n* = 20) from 10 days post-fertilization (dpf) until hatching. The vertical line in A indicates the time point when embryos were either too large to measure because they started overlapping themselves within the egg or the neonate had hatched. The vertical line in B represents the time point when the yolk was too small to measure and/or the embryo was too large and overlapping the yolk, thus precluding measurement.

The daily growth rate of embryos exposed to elevated CO_2_ did not differ significantly when compared with their control counterparts (*F*_1,4_=2.817, *P* = 0.169; Fig. 2A). Embryos from both treatments exhibited similar growth curves, with a slow increase in growth for the first 40 days and more rapid growth in the following days (Fig. 2A). Embryo growth depended on their dpf (a time factor) in the control group and CO_2_ treatment, as expected. There was no effect of embryo origin or tank, and no interactions were detected between any of the variables (supplementary material Table S1).

### Yolk consumption

The average amount of yolk used (percentage remaining, starting from 100%) by embryos did not differ significantly over time between control animals and those reared in elevated CO_2_ conditions (*F*_1,4_=0.172, *P* = 0.6995; Fig. 2B). There was no interaction between treatment, time, origin or tank (supplementary material Table S1). Although not significant, once embryos in the elevated CO_2_ treatment reached 41 dpf, they had used around 11% more yolk than their control counterparts (Fig. 2B).

### Movement

**Figure 3:**
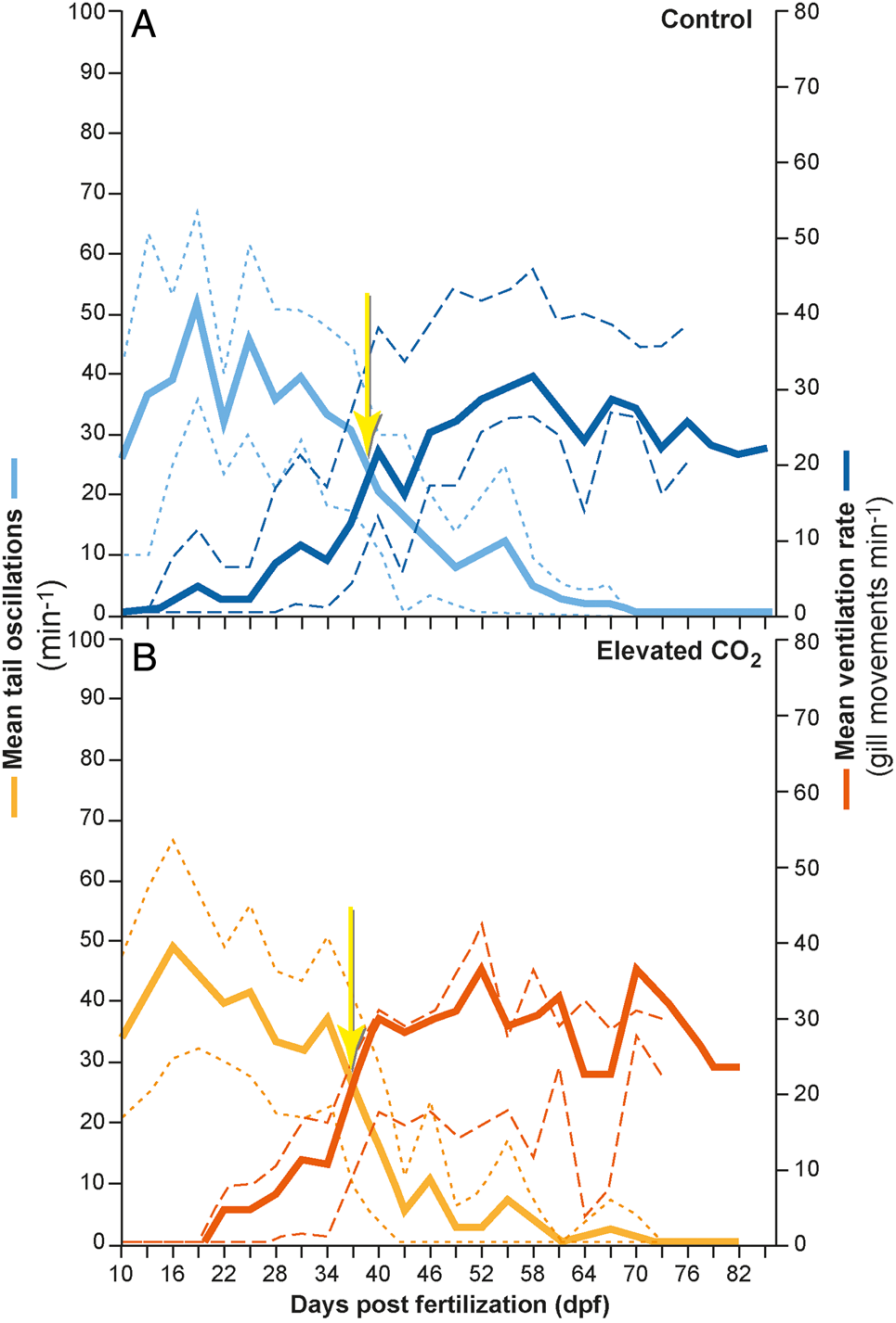
Mean (95% confidence interval) tail oscillations (per minute; primary *y*-axis) and ventilation rate (gill movements min^−1^; secondary *y*-axis) of embryos reared in control conditions (**A**; 420 μatm; *n* = 16) or in elevated CO_2_ conditions (**B**; 940 μatm; *n* = 20) over time until hatching.

Tail oscillation rates decreased over time, but ventilation rates increased over time in embryos from the control and elevated CO_2_ groups (Fig. 3). There were no significant differences in these trends between treatments (tail oscillation rates *F*_1,4_ = 0.001, *P* = 0.938; and ventilation rates *F*_1,4_ = 0.656, *P* = 0.464); however, there was a point in time when tail oscillation rate and ventilation rate trends intersected, ∼40 dpf in the control animals (Fig. 3A) and slightly earlier, ∼37 dpf, in animals reared in elevated CO_2_ (Fig. 3B). This intersection coincided with the average time post-fertilization for the gill slits to appear. The time of intersection was not significantly different between the control elevated CO_2_ groups, nor was the point (in dpf) at which gill slits appeared.

### Survival

**Figure 4:**
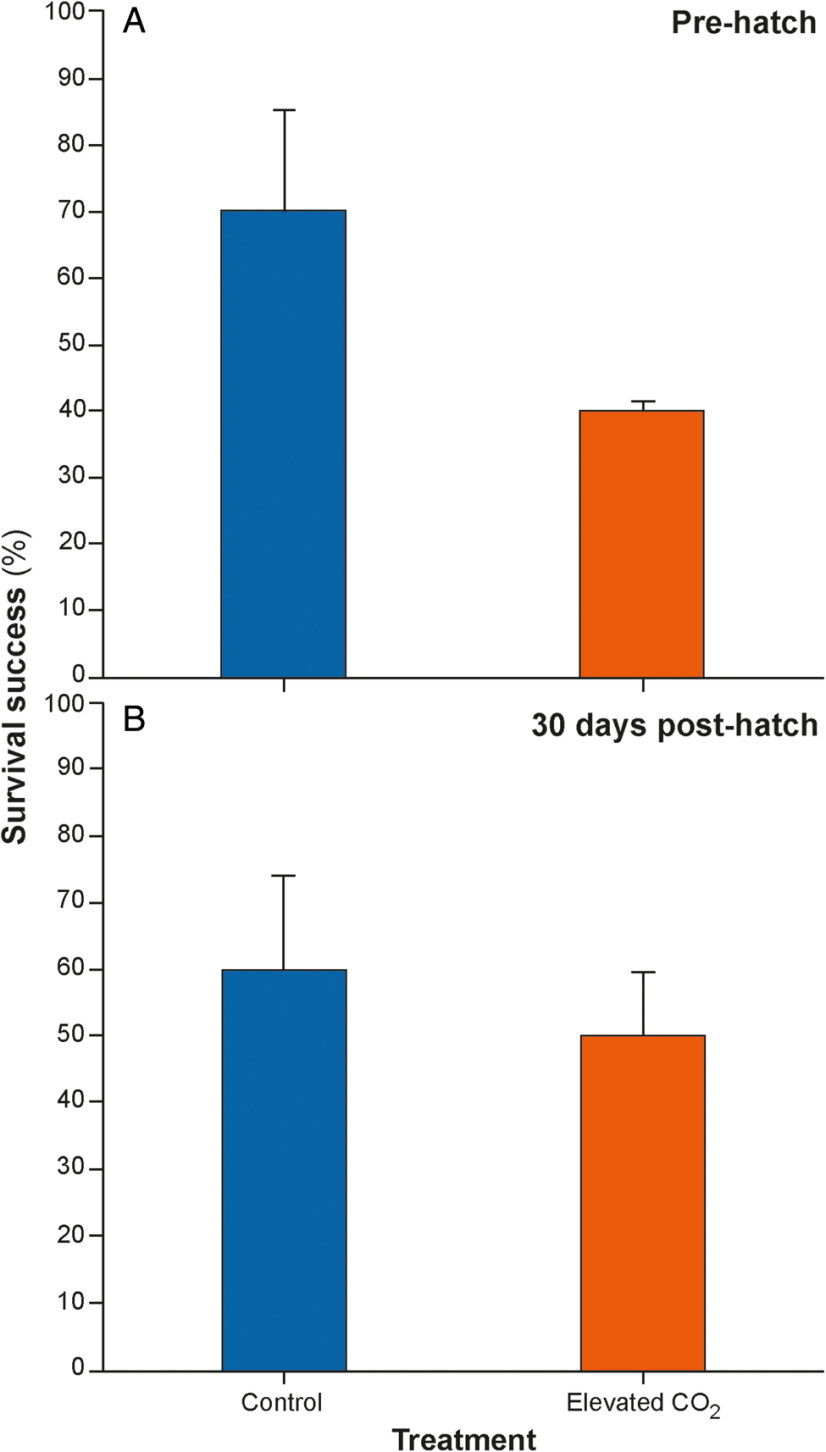
Survival (expressed as a percentage) in embryos reared in control conditions (420 μatm) and in elevated CO_2_ (940 μatm). (**A**) Survival of embryos from fertilization until hatching (*n* = 36). (**B**) Survival of neonates for 30 days post-hatch (*n* = 11; control *n* = 7 and elevated CO_2_*n* = 4).

**Figure 5:**
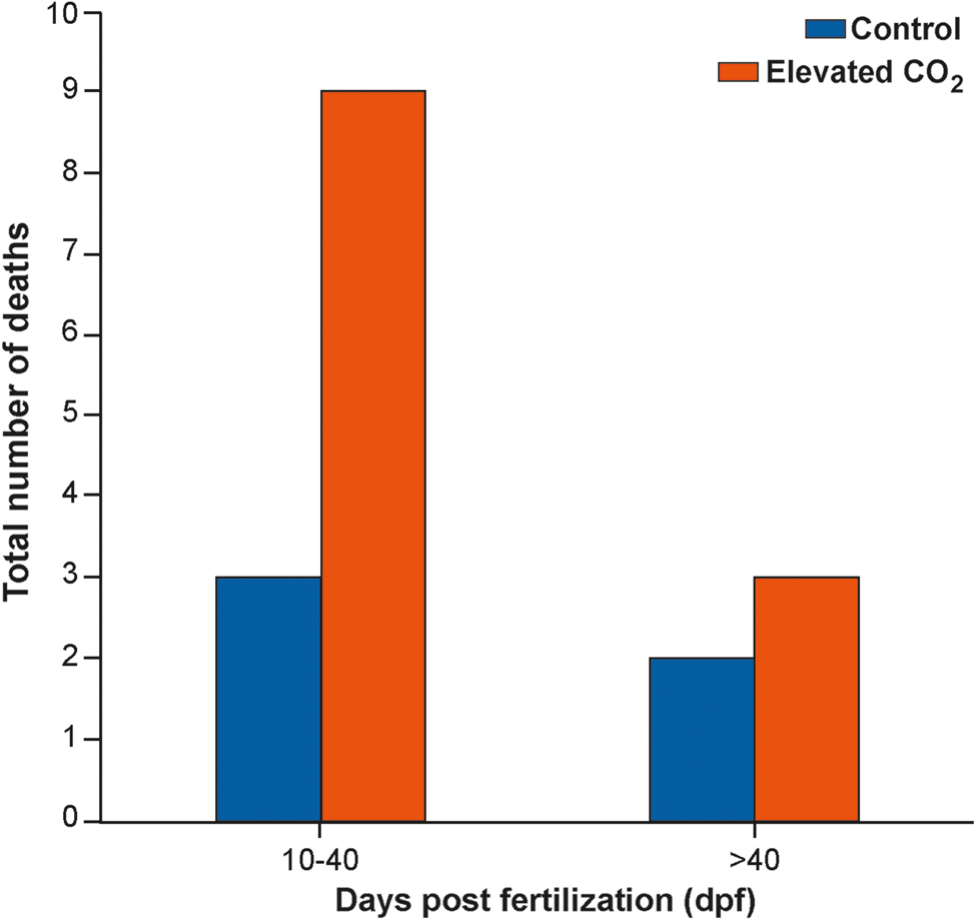
Total number of observed deaths distributed by early development (10–40 dpf) and later embryonic development (>40 dpf) in embryos reared in control conditions (420 μatm) and in elevated CO_2_ (940 μatm).

There was no significant difference in hatching success between control embryos (70.0 ± 15.3%) and embryos reared in elevated CO_2_ (40.1 ± 1.6%; χ^2^ = 2.95, d.f. = 1, *P* = 0.086; Student's *t*-test = 1.946, d.f. = 4, *P* = 0.123); however, hatching was ∼75% more likely in embryos reared in control compared with elevated CO_2_ conditions (Fig. 4A). For control animals, four of the five deaths occurred early in development (between 10 and 40 dpf), and for embryos reared in elevated CO_2_, nine of the 12 deaths occurred between 10 and 40 dpf (Fig. 5). A similar trend was observed in survival post-hatch, where 60.0 ± 13.9% of the embryos that successfully hatched in control conditions survived for at least 30 days compared with 50.0 ± 9.6% of the embryos that successfully hatched in elevated CO_2_ conditions (Fig. 4B). Survival 30 days post-hatching was 20% more likely in control animals than in elevated CO_2_ animals, but this trend also was not statistically significant (χ^2^ = 0.015 d.f. = 1 *P* = 0.901; Student's *t*-test = 0.592, d.f. = 4, *P* = 0.586).

## Discussion

The capacity for marine organisms to acclimate and adapt to changing ocean conditions has become one the most pressing issues in marine science (Doney *et al*., 2009a). Owing to the important role played by sharks as predators in marine ecosystems, it is of the utmost importance to study and gain a better understanding of their ability to maintain homeostasis when exposed to changing ocean conditions (Chin *et al*., 2010; Renshaw *et al*., 2012). The present study has highlighted the ability of *H. ocellatum* embryos to tolerate end-of-century pCO_2_ and therefore ocean acidification conditions. Findings here suggest that growth rates and yolk consumption rates, as well as ventilation and tail oscillation rates are unchanged in elevated CO_2_ conditions compared with present-day (control) conditions. These data suggest that *H. ocellatum* could tolerate end-of-century CO_2_ levels without negative effects on early life-history stages. The epaulette shark's tolerance of elevated CO_2_ conditions may be related to specialized adaptations that enable them to survive extreme fluctuations in oxygen levels in their habitat (Mulvey and Renshaw, 2000; Renshaw *et al*., 2002; Speers-Roesch *et al*., 2012).

Although overall unaffected by developing in elevated CO_2_, hatching success and post-hatching survival was approximately 43 and 23% higher, respectively, in control-reared embryos than in embryos reared in elevated CO_2_. Although these trends were not statistically significant, probably as a result of large inter-animal variance, this is something that could be targeted in future studies. Taken together, findings from the present study suggest that elevated CO_2_, although not affecting selected metrics of healthy development, may still have detrimental effects on some aspects of development that are important for hatching and survival but were not examined in this study.

### Embryo growth and development

Growth rates were similar between embryos exposed to the control conditions and the CO_2_ treatment (Fig. 2A), which may not be surprising given previous evidence of physiological and behavioural tolerance of elevated CO_2_ in adult epaulette sharks (Heinrich *et al*., 2014, 2015). A similar result was also observed in the brown-banded bamboo shark (*C. punctatum*), where growth rates and yolk consumption rates did not differ between embryos reared in present-day and end-of-century pCO_2_ levels (Rosa *et al*., 2014). If shark embryos are challenged by a need to regulate acid–base/ion balance in elevated CO_2_, then animals may need to expend more energy to regulate pH and ion homeostasis, thus potentially diverting energy away from important processes, such as growth, which could result in smaller embryos (Renshaw *et al*., 2012; Skomal and Mandelman, 2012). As growth rates were similar between the control animals and those reared in elevated CO_2_ conditions, it is possible that *H. ocellatum* embryos were able to acid–base/ion regulate efficiently without expending additional energy, which is consistent with findings for mature *H. ocellatum* (Heinrich *et al*., 2014, 2015).

Maintaining healthy growth rates is important for K-selected species, such elasmobranchs, as they invest a lot of energy in fewer offspring to ensure greater survival; size is important because large neonates may be less susceptible to predation than small neonates (Pianka, 1970; Ebert *et al*., 2008). Neonate size is especially important to oviparous elasmobranch species that develop in egg cases without maternal care. It is important that no extra time is spent developing than is necessary. Embryos *in ovo* are unable to choose favourable environmental conditions or escape from predators; however, maternal instincts command female elasmobranchs to deposit eggs in favourable locations whilst still being site attached (Hamlett, 2005; Rodda and Seymour, 2008; Amsler *et al*., 2015). The maternal role in egg deposition and how this may change with alterations in water quality and climate change will be an avenue to explore in future studies.

### Yolk consumption

The rate of yolk consumption was not significantly different between embryos reared in control and elevated CO_2_ conditions, indicating that animals from both groups were taking up similar amounts of nutrients over time. An interesting trend can be seen between around 41 dpf, when embryos reared in elevated CO_2_ had already consumed an average of 11% more yolk than control-reared embryos. However, embryos reared in elevated CO_2_ were slightly smaller at the same time period when compared with control animals (Figs 1 and 2A), indicating that some growth might be lost. This result was in agreement with the results presented for the brown-banded bamboo shark (Rosa *et al*., 2014) and little skate (Di Santo, 2015); both those species exhibited average daily yolk consumption rates that differed between treatment temperatures but not with elevated CO_2_. This non-significant difference is an important finding in the early life stages of any oviparous marine species, because the yolk sac is the sole energy reserve and key to nourishing the metabolic activities essential for growth and survival (Rodda and Seymour, 2008). Conclusions such as these further support the hypothesis that benthic mesopredators occupying similar ecological niches associated with habitats that are known to have regular CO_2_ fluctuations may be better equipped to survive embryonic development through to hatching in future ocean conditions.

### Movement and ventilation

Tail oscillations represent an important activity for elasmobranch embryos because the tail moves water around the egg and over the embryo's skin, which facilitates gas exchange (e.g. oxygen) and perhaps even acid–base/ion regulation during early development (Ballard and Lechenault, 1993; Rodda and Seymour, 2008). The gills are not fully developed until ∼40 dpf, and before this time the animal must depend on cutaneous gas exchange and, possibly, ion transport. Tail oscillations ensure constant mixing of the water around the embryo and gas exchange with the external environment via diffusion through the egg case. It is counterintuitive that tail oscillations of embryos reared in elevated CO_2_ conditions did not differ from those of embryos reared in control conditions because it would be expected that developing epaulette sharks in elevated CO_2_ would have needed to increase tail oscillation rates to replace high-CO_2_ water more frequently than their control counterparts. Tail oscillation rates did decrease over time, but this was probably because of the embryos growing larger and occupying more space within the egg case, resulting in less room for movement, which has been documented in other studies (Ballard and Lechenault, 1993; Rodda and Seymour, 2008). The reduction in tail oscillation rates could also be related to gill development. Once gills are fully developed, there is less urgency to mix the water surrounding the embryo to aid cutaneous diffusion, because the gills become a more efficient mechanism for gas exchange and acid–base/ion regulation during the latter stages of development (Pelster and Bemis, 1992; Ballard and Lechenault, 1993).

Gills were first observed moving/ventilating at ∼40 dpf in embryos reared in control conditions and 37 dpf for embryos reared in elevated CO_2_ conditions. The developmental milestone of gill development occurred at the midpoint of embryonic development. This was comparable to results from Harahush *et al*. (2007) on the tropical oviparous brown-banded bamboo shark (*C. punctatum*), which also develops the gills around the midpoint of embryonic development (54% of incubation), and results from Rodda and Seymour (2008) on the Port Jackson shark (*H. portusjacksoni*), which also develops the gills by the midpoint of embryonic development. Gill movements are a good proxy for ventilation rates and may have been expected to differ in elevated CO_2_ conditions because ventilatory adjustments are one means through which gas exchange is maintained (Gilmour, 2001), but they did not differ in this study.

It is understood that fish (including elasmobranchs) do not hyperventilate to compensate for elevated blood CO_2_ in the way that we understand for air-breathing animals (Heuer and Grosell, 2014). Metabolic (vs. respiratory) compensation for an acidosis would presumably be less energetically costly than changes in gill ventilation (Heuer and Grosell, 2014). Therefore, if changes in ventilation rates or amplitude were observed, as in the big skate (*Raja ocellata*) that increases ventilation rates by 3-fold in elevated CO_2_ (Graham *et al*., 1990), they could be related to other functions, such as ion balance or immune function. The hyperventilatory response has been most widely researched in animals upon exposure to CO_2_ levels 10–50 times greater than what was used here, and the response is also likely to vary according to species (Heuer and Grosell, 2014) and, probably, life stage. Furthermore, during the embryonic life stages of aquatic animals, the driving force for gill development has been demonstrated to be acid–base/ion regulation, well before the need to compensate for O_2_ limitations (Fu *et al*., 2010; Brauner and Rombough, 2012). This may have been the case in the present study as well.

### Survival

Embryos reared in elevated CO_2_ exhibited a 22% chance of survival, whereas control-reared embryos exhibited a 31% chance of survival. Overall survival in tropical bamboo shark (*C. punctatum*) embryos was determined to be unaffected by elevated CO_2_ (Rosa *et al*., 2014). However, Baumann *et al*. (2012) and Forsgren *et al*. (2013) found decreased survival of teleost eggs in elevated CO_2_ conditions and hypothesized that is could be due to the extra costs associated with acid–base/ion regulation. Although the survival rates in the present study seemed low, particularly in the elevated CO_2_ treatment, numerous studies have found mortality of eggs in the wild or in the laboratory to be between 20 and 80% (Chen and Liu, 2006; Harahush *et al*., 2007; Griffiths *et al*., 2012). Elevated CO_2_ could be further affecting embryo survival and hatching success because of the added energetic costs not detected by our measurements.

Another interesting trend to note is that most pre-hatching mortality occurred before embryos reached 40 dpf (Fig. 5), a time that corresponded to gill development in this species. Of the 17 deaths observed in this study, most occurred between 10 and 40 dpf and, of those, most were in embryos reared in elevated CO_2_ (Fig. 5). Elevated CO_2_ could cause the embryo to divert energy away from growth to ensure that gas exchange and acid–base/ion regulation are maintained (Renshaw *et al*., 2012; Skomal and Mandelman, 2012). This is highly plausible as embryos would be much more susceptible prior to gill development, because the gills are the major organ responsible for acid–base balance in elasmobranchs (Tresguerres *et al*., 2005). In teleosts reared in elevated CO_2_ conditions, modifications in proteins related to energy generation have been documented (De Souza *et al*., 2014). The need for acid–base and ion regulation in conditions of low pH has also been shown to increase energetic cost in other studies (Deigweiher *et al*., 2008; Melzner *et al*., 2009). However, this has not yet been demonstrated in elasmobranchs.

If an embryo died before hatching but their egg case still had an intact mucous plug, no fungal infections were observed; in contrast, fungal infections were common in embryos where the mucous plug had already dissolved from the egg case. Once fungus appeared, egg cases started to appear white in colour and started to be smelly. Embryos usually died within 24 h. This white fungus has been reported in early development in other elasmobranch species but has not yet been identified (Harahush *et al*., 2007; Payne, 2012).

Neonate survival at 30 days post-hatch was 50% in the animals reared in elevated CO_2_ conditions and 64% in the animals reared in control conditions, which was similar to the 54% survival rate determined by Rosa *et al*. (2014) in sharks reared in elevated CO_2_. The differences in mortality rates between control and elevated CO_2_-reared sharks, although non-significant, could have been due to the stress responses of the embryos and downstream effects on other systems, such as immune function. If more energy is being used to maintain homeostasis, less energy is available for growth and/or development of other important processes. Survival may also have improved post-hatch because neonates were transplanted into control conditions which, given the non-significant differences in survival rates, suggests that there were no lasting effects of being reared in elevated CO_2_ conditions as embryos.

### Overall implications and future studies

Development, post-fertilization, is the beginning of all vertebrate life; without achieving the milestones laid down in early development, individuals are less likely to survive and reproduce to guarantee the next generation. Although the vulnerability of elasmobranchs to climate change is thought it to be species specific, whether indirectly or directly, elevations in environmental CO_2_ are predicted to result in some deleterious consequences for elasmobranchs in the future (Chin *et al*., 2010). This study highlights that *H. ocellatum* may have adaptive mechanisms that confer tolerance to elevated CO_2_ conditions during early development, as well as into adulthood, as has already been confirmed (Heinrich *et al*., 2014, 2015). This may be the case for other mesopredator species as well. In the case of *H. ocellatum*, tolerance may be linked to the time at which the gills fully form, and future studies can focus on this important milestone in development. However, tolerance does not always come without trade-offs. Although several key developmental measures were not significantly affected by elevated CO_2_, future studies could increase replication and focus on some of the interesting trends revealed here but not specifically examined. For example, the higher level of mortality observed during development, especially before gill development, and post-hatch in animals reared in elevated CO_2_ could be key and may prove even more deleterious in oviparous elasmobranch species that, unlike the epaulette shark, do not regularly experience diurnal fluctuations in O_2_ and CO_2_ in their local environments.

At the ecosystem level, mesopredators such as the epaulette shark provide a link between apex predators and the lower trophic levels (Vaudo and Heithaus, 2011; Yick *et al*., 2012). If some populations of elasmobranch mesopredators possess the capacity to acclimate to challenging environmental conditions and/or the necessary adaptations that confer tolerance to such conditions (Wise *et al*., 1998; Nilsson and Renshaw, 2004; Heinrich *et al*., 2014, 2015) and others do not (Rosa *et al*., 2014; Di Santo, 2015; Pistevos *et al*., 2015), this poses some interesting questions regarding population and range expansion and the ecosystem-level ramifications. Woodland *et al*. (2011) found that some populations of mesopredatory elasmobranchs that occupy shallow coastal waters share similar ecological niches to piscivorous fishes. However, these elasmobranchs, although abundant, do not seem to affect surrounding teleost populations, mainly as a result of low competition for resources. Likewise, Navia *et al*. (2010) modelled mesopredatory elasmobranch populations and concluded that expansions in sicklefin smooth-hound shark (*M. lunulatus*) and longtail stingray (*Dasyatis longa*) populations would have little to no immediate effects on prey abundance, further supporting the notion that an expansion in mesopredatory elasmobranch populations would have little effect on the lower trophic levels competing for similar resources. Although few studies have examined the effects of elevated CO_2_ on the ecological role of mesopredatory elasmobranchs, current knowledge suggests that increases in *H. ocellatum* populations would have little to no effect on lower trophic level teleosts. Indeed, differential responses to climate change-related stressors, such as elevated CO_2_ and ocean acidification, will result in winners, such as *H. ocellatum*, and losers at the species level, but population- and ecosystem-level studies are required to elucidate the broader impacts.

## Supplementary material

Supplementary material is available at *Conservation Physiology* online.

## Funding

This research was supported by funding from the Australian Research Council (ARC) Discovery, early career grant [PDE150101266] and ARC Super Science Fellowship [FS100100088], both to J.L.R., and through a grant from Griffith University (Gold Coast) Climate Change Response Group to G.M.C.R. and J.L.R.

## Supplementary Material

Supplementary Data
