# Supplementary material for: Will ocean acidification affect the early ontogeny of a tropical oviparous elasmobranch (Hemiscyllium ocellatum)?
Source: Conserv Physiol. 2016 Mar 4;4(1):cow003. doi: 10.1093/conphys/cow003 (PMC4784014; doi:10.1093/conphys/cow003)
Supplement: Supplementary Data [file cow003supp.zip › cow003supp.docx]

**Supplementary Materials**

**Table S1:** Statistical Analysis (ANOVA) outputs including df, F-values and P-values from proportional growth, proportion of yolk consumed, tail oscillation rates and ventilation rates for sharks reared under control or elevated CO_2_ treatments. Origin indicates whether eggs were supplied from Cairns Marine or SeaWorld, and dpf is days post fertilization.

|  | df | F-value | P-value |
| --- | --- | --- | --- |
| **Embryo Development** |  |  |  |
| Treatment | 1 4 | 2.8169 | 0.1686 |
| Origin | 1 21 | 22.5593 | **0.0001** |
| dpf | 1 290 | 48.0018 | **<0.0001** |
| Treatment:Origin | 1 21 | 2.5417 | 0.1258 |
| Treatment: dpf | 1 290 | 0.5240 | 0.4697 |
| Origin:dpf | 1 290 | 0.8303 | 0.3629 |
| Treatment:Origin:dpf | 1 290 | 1.2671 | 0.2612 |
| **Yolk Usage** |  |  |  |
| Treatment | 1 4 | 0.17214 | 0.6995 |
| Origin | 1 21 | 3.60459 | 0.0714 |
| dpf | 1 279 | 1.34547 | 0.2471 |
| Treatment:Origin | 1 21 | 2.15007 | 0.1574 |
| Treatment: dpf | 1 279 | 1.16121 | 0.2821 |
| Origin:dpf | 1 279 | 0.00032 | 0.9856 |
| Treatment:Origin:dpf | 1 279 | 3.90884 | **0.0490** |
| **Tail Oscillation Rate** |  |  |  |
| Treatment | 1 4 | 0.0001 | 0.9938 |
| Origin | 1 21 | 4.3600 | **0.0492** |
| dpf | 1 376 | 233.6285 | **<0.0001** |
| Treatment:Origin | 1 21 | 1.7616 | 0.1987 |
| Treatment: dpf | 1 376 | 1.0506 | 0.3060 |
| Origin:dpf | 1 376 | 9.8879 | **0.0018** |
| Treatment:Origin:dpf | 1 279 | 0.0024 | 0.9610 |
| **Ventilation Rate** |  |  |  |
| Treatment | 1 4 | 0.6525 | 0.4645 |
| Origin | 1 20 | 1.7762 | 0.1976 |
| dpf | 1 369 | 361.7696 | **<0.0001** |
| Treatment:Origin | 1 20 | 0.1040 | 0.7504 |
| Treatment: dpf | 1 369 | 0.0448 | 0.8326 |
| Origin:dpf | 1 369 | 9.5492 | **0.0022** |
| Treatment:Origin:dpf | 1 369 | 2.8153 | 0.0942 |
